# Nerve Prostration, and Other Functional Disorders of Daily Life

**Published:** 1892-03

**Authors:** 


*Nerve Prostration, and other Functional Disorders of Daily Life.* By ROBSON ROOSE, M.D., LL.D., F.C.S. Second Edition. Pp. xxii., 671. London: H. K. Lewis. 1891.

A second edition calls for little comment. The book is growing in proportion to the growth in experience of its author, who interprets the term "functional" on very liberal principles, and has produced nearly seven hundred pages, in excellent type, on the best of paper, teeming with the matured convictions of one who has every claim to write with authority on the little ills of every-day life and the "little health of ladies." The tired practitioner may take it up at odd moments, and his patients will probably benefit thereby.

